# Slit-Lamp Management of Silicone Oil-Induced Pupillary Block After Vitrectomy

**DOI:** 10.7759/cureus.95016

**Published:** 2025-10-20

**Authors:** Keigo Takagi, Kazunobu Sugihara, Kana Murakami, Masaki Tanito

**Affiliations:** 1 Department of Ophthalmology, Shimane University Faculty of Medicine, Izumo, JPN

**Keywords:** acute angle-closure glaucoma, angle-closure glaucoma, ophthalmic viscosurgical device, pupillary block, pupillary block glaucoma, silicone oil, silicone oil in anterior chamber, silicone oil migration, slit-lamp procedure, vitrectomy

## Abstract

Silicone oil (SO) is commonly used as a tamponade agent in vitreoretinal surgery for complex retinal detachments, but its migration into the anterior chamber (AC) can cause pupillary block and secondary angle-closure glaucoma (ACG). We report a 51-year-old Japanese man with proliferative diabetic retinopathy who underwent combined phacoemulsification and pars plana vitrectomy with SO tamponade for tractional retinal detachment. On postoperative day 1, SO was observed in the AC with normal intraocular pressure (IOP) and anterior chamber depth; however, on day 2, he developed pupillary block with a flattened AC and IOP of 60 mmHg. At the slit lamp, a dispersive ophthalmic viscosurgical device was injected through a corneal side port, and the iris was depressed posteriorly with a blunt cannula, allowing aqueous humor to re-enter the AC. A transcorneal peripheral iridotomy was then performed with a microvitreoretinal blade, resulting in the resolution of the pupillary block and normalization of IOP. This case highlights a practical outpatient technique that enables an effective management of SO-induced pupillary block at the slit lamp, avoiding the risks of supine surgical intervention or premature SO removal and preserving the tamponade effect.

## Introduction

Silicone oil (SO) is widely used as a tamponade agent in vitreoretinal surgery for severe conditions such as rhegmatogenous retinal detachment (RRD), proliferative vitreoretinopathy (PVR), and proliferative diabetic retinopathy (PDR) [1-3]. While retained in the eye, SO provides a sustained tamponade effect and is important in refractory cases. However, complications such as corneal endothelial damage, cataract formation, unexplained visual loss, and secondary glaucoma have been reported [4-6]. Among these, postoperative ocular hypertension (OH) and secondary glaucoma can arise through various mechanisms [7]. Previous studies have shown that migration of SO into the anterior chamber (AC) may cause pupillary block and angle-closure glaucoma (ACG) [7]. We present a case of SO-induced pupillary block with secondary ACG and describe its effective management and clinical course.

## Case presentation

A 51-year-old Japanese man presented to a local ophthalmologist with decreased vision in his right eye (RE) for two months. He was diagnosed with tractional retinal detachment (TRD) due to PDR in the RE and was referred to our hospital.

**Figure 1:**
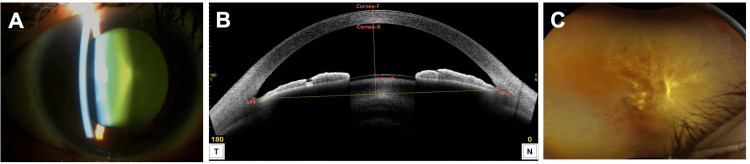
Slit-lamp examination (A), AS-OCT (B), and ultra-widefield fundus photograph (C) of the right eye. (A) An anterior segment photograph shows a nuclear cataract. (B) AS-OCT reveals a normal anterior chamber depth. Cornea-F: front surface of the cornea; Cornea-B: back surface of the cornea; Lens-F: front surface of the lens; AR1: angle recess 1; AR2: angle recess 2. (C) Ultra-widefield fundus photograph demonstrates tractional retinal detachment secondary to fibrovascular proliferation. AS-OCT: Anterior segment optical coherence tomography.

On initial examination, slit-lamp biomicroscopy revealed nuclear cataracts in both eyes (Figure 1A). Anterior segment optical coherence tomography (AS-OCT, Casia 2, Tomey Corporation, Nagoya, Japan) showed a normal AC depth in the RE (Figure 1B). Fundus examination revealed a fibrovascular membrane on the nasal side of the optic disc with TRD extending ~120° from the superonasal to the inferior quadrant (Figure 1C). The left eye (LE) had diabetic macular edema. Retinal photocoagulation scars were present in all quadrants from the equator to the periphery in both eyes. Best-corrected visual acuity (BCVA) was 0.1 in the RE and 0.8 in the LE. Intraocular pressure (IOP) was 16 mmHg in the RE and 20 mmHg in the LE. Combined cataract surgery and vitrectomy in the RE was scheduled one month later. An intravitreal injection of aflibercept (Eylea, Santen Pharmaceuticals, Osaka, Japan) was administered one day before surgery.

The patient underwent uneventful phacoemulsification, with no evidence of zonular weakness, followed by 27-gauge pars plana vitrectomy (PPV). Two iatrogenic retinal breaks occurred intraoperatively in the inferonasal retina. The surgery was completed with SO tamponade. Before concluding, the AC became shallow. Injection of a dispersive ophthalmic viscosurgical device (OVD) (Shellgan, Santen Pharmaceuticals, Osaka, Japan) restored AC depth, but SO was observed migrating into the AC. Iridectomy was not performed due to concern that further AC manipulation could promote additional SO migration.

**Figure 2:**
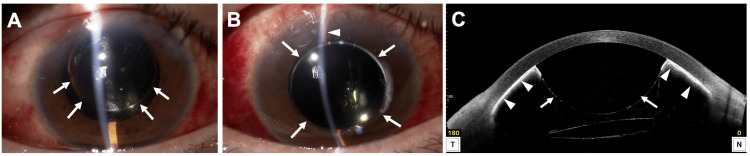
Slit-lamp examination (A, B) and AS-OCT (C) of the right eye after phacovitrectomy. (A) An anterior segment photograph on the first postoperative day shows silicone oil (SO) in the anterior chamber (AC, arrow) and a normal AC depth. (B) An anterior segment photograph on the second postoperative day shows centrally migrated SO in the AC (arrow) and a flat AC (arrowhead). (C) AS-OCT on the second postoperative day shows complete pupillary blockage by migrated SO (arrow) and adhesion of the entire iris to the posterior cornea (arrowheads). AS-OCT: Anterior segment optical coherence tomography.

On postoperative day 1, IOP in the RE was 15 mmHg, the cornea was clear, AC depth was normal, and SO was observed in the AC (Figure 2A). On day 2, IOP increased to 60 mmHg and the AC was flat (Figure 2B). AS-OCT revealed a pupillary block caused by SO migration into the AC (Figure 2C). The other possible mechanisms such as aqueous misdirection, choroidal expansion/effusion and silicone oil overfill were all excluded by AS-OCT findings, based on the normal position of the ciliary body and IOL.

**Figure 3:**
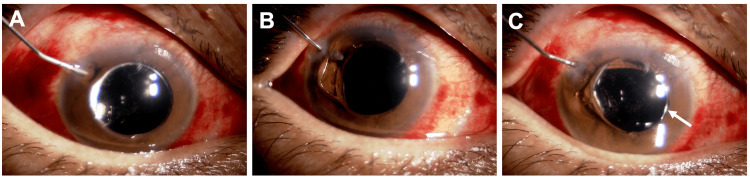
Release of SO-induced pupillary block by iris depression with injection of an ophthalmic viscosurgical device (OVD) into the AC. (A) A blunt cannula is introduced through a corneal side port, and OVD is injected over the iris. (B) The iris is depressed using the blunt cannula, allowing aqueous humor to flow from the posterior chamber into the AC. (C) The AC is reformed. Migrated SO is seen in the AC (arrow). AC: Anterior chamber; SO: silicone oil.

**Figure 4:**
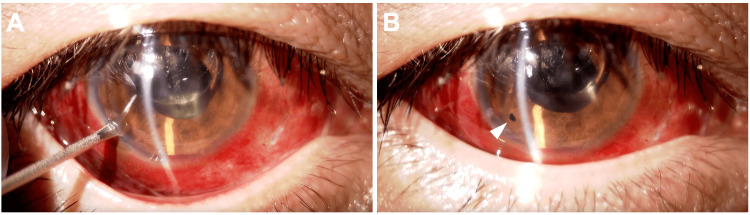
Iridotomy performed using a microvitreoretinal (MVR) blade. (A) An MVR blade is inserted at the 7 o’clock position of the corneal periphery, and the iris is penetrated. (B) The completed iridotomy is confirmed (arrowhead).

To relieve the block, a procedure was performed at the slit lamp with the patient in a sitting position. Before the procedure, disinfection with povidone-iodine and topical anesthesia with oxybuprocaine were performed. Neither a drape nor a lid speculum was used. A dispersive OVD was injected through an existing corneal side port at the 10 o’clock position (Figure 3A, Video 1), and the iris was depressed posteriorly with a blunt cannula (Figure 3B). Aqueous humor re-entered the AC, restoring its depth immediately (Figure 3C). To prevent recurrence, a transcorneal peripheral iridotomy was created in the inferotemporal quadrant using a 20-gauge microvitreoretinal (MVR) blade (Mani, Inc., Tochigi, Japan) (Figure 4A, B, Video 2). After the procedure, the patient continued the pre-existing regimen of 1.5% levofloxacin and 0.1% betamethasone four times daily.

**Video 1:** Release of SO-induced pupillary block by injection of an ophthalmic viscosurgical device into the anterior chamber. SO: Silicone oil.

**Video 2:** Iridotomy performed using an microvitreoretinal (MVR) blade.

**Figure 5:**
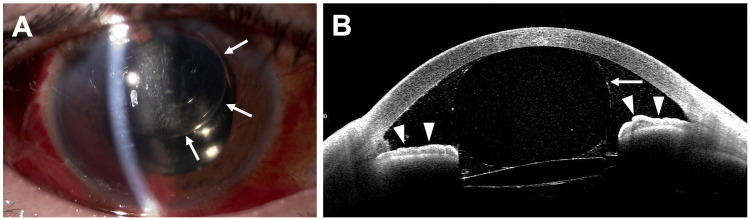
Slit-lamp examination (A) and AS-OCT (B) of the right eye three days after surgery. (A) An anterior segment photograph shows a normal AC depth. Migrated SO is present in the AC (arrow). (B) AS-OCT demonstrates resolution of the pupillary block (arrowheads). Migrated SO remains in the AC (arrow). AS-OCT: Anterior segment optical coherence tomography; AC: anterior chamber; SO: silicone oil.

Thereafter, the AC depth normalized, the pupillary block resolved, and IOP decreased to 12 mmHg (Figure 5A, B). One and a half months later, the patient underwent SO removal by 25-gauge PPV.

## Discussion

SO is biocompatible with ocular tissues and is used in vitreoretinal surgery for refractory retinal diseases [8]. It provides long-term tamponade by maintaining adhesion between the neurosensory retina and the retinal pigment epithelium. Elevated IOP following SO tamponade may occur through open-angle or angle-closure mechanisms [9]. Open-angle causes include overfilling, SO emulsification with inflammation, exacerbation of pre-existing glaucoma, and SO droplet infiltration into the trabecular meshwork [10]. Angle-closure causes include extensive peripheral anterior synechiae and pupillary block due to AC migration of SO [9,11]. In this case, marked OH occurred due to pupillary block.

Although rare, pupillary block can occur after vitreoretinal surgery with SO. The unique feature of our case is the description of a simple outpatient procedure performed at the slit lamp with the patient in a sitting position. The technique first relieves the block by depressing the iris posteriorly, thereby overcoming the surface tension of SO, followed by iridotomy to establish an alternative aqueous pathway. At least in this case, the method we report was effective. In our case, yttrium aluminum garnet (YAG) laser iridotomy could be considered as one possible management option. However, performing iridotomy requires the use of a contact lens, and AC compression by the lens may potentially cause further SO migration. Moreover, our method has the advantage of not requiring any special laser equipment, and it can be applied in situations where laser treatment is not feasible due to severe corneal opacity. Operating room intervention is an alternative; however, the supine position may increase the risk of further SO migration into the AC because SO has a lower specific gravity than aqueous humor [12]. Another option, SO removal, risks inadequate vitreous cavity volume and compromised tamponade effect [13]. Thus, the described method offers a valuable outpatient solution.

SO can migrate into the AC due to zonular weakness, even in phakic or pseudophakic eyes [14]. Once migrated, it induces pupillary block, leading to a shallow AC, circumferential iridotrabecular contact, and OH. In this case, we suspect partial zonular damage during maneuvers such as scleral indentation or vitreous shaving. Pupillary block from SO tamponade is more common in aphakic patients; therefore, prophylactic inferior iridectomy should be considered [15]. In this case, SO migration was already observed at the end of surgery, suggesting zonular weakness and indicating that an intraoperative iridotomy with a vitreous cutter might have been preferable. Although intraoperative iridotomy was not performed due to concern about worsening SO migration in the supine position, the method described here could have been applied intraoperatively to prevent postoperative block.

## Conclusions

This case demonstrates an effective technique for managing SO-induced pupillary block after vitrectomy. Although relatively rare, SO-related ACG can also occur in pseudophakic eyes. In cases where postoperative ACG develops, the slit-lamp technique described here may provide a simple and effective option for resolution.
